# *Acanthopanax senticosus* total flavonoids alleviate lipopolysaccharide-induced intestinal inflammation and modulate the gut microbiota in mice

**DOI:** 10.1042/BSR20212670

**Published:** 2022-02-07

**Authors:** Xiaoya Wang, Xinyu Zhang, Jianqing Su, Xiuling Chu

**Affiliations:** College of Agronomy, Liaocheng University, Liaocheng 252000, China

**Keywords:** gut microbiota, inflammation, lipopolysaccharides

## Abstract

Here, we study the therapeutic effect of *Acanthopanax senticosus* total flavonoids (ASTFs) using a mouse intestinal inflammation model. The inflammation model used in the present study was developed through lipopolysaccharide (LPS) treatment of mice. The experimental mice were divided into a control group, model group (10 mg/kg LPS), dexamethasone group (1 mg/kg DEX) and ASTF low-, medium- and high-dosage groups (200, 400 and 800 mg/kg, respectively). The morphological and structural changes in the ileum, jejunum and duodenum were observed using HE staining. The number of intestinal goblet cells (GCs) was calculated based on PAS staining. The contents of interleukin (IL)-1β, IL-6, prostaglandin E2 (PGE2) and tumor necrosis factor α (TNF-α) were determined using enzyme-linked immunosorbent assay (ELISA) and the related mRNA expression level were measured by RT-PCR. The protein expression levels of Toll-like receptor 4 (TLR4), MyD88, p65 and p-p65 were measured using Western blotting. In addition, the 16S rRNA sequences of bacterial taxa were amplified and analyzed to assess changes in the intestinal microbes of LPS-induced mice and also in response to regulation by ASTF. Following intervention with ASTF, different therapeutic effects were shown according to the various dosages tested, all of which resulted in improved intestinal morphology and an increased number of intestinal GCs, while the contents of IL-1β, IL-6, PGE2 and TNF-α and the related mRNA expression level were significantly reduced. The TLR4, MyD88 and p-p65/p-65 protein expression levels were also significantly reduced. In addition, 16S rRNA sequencing results show that LPS disrupts the structure of mouse gut microbes, though we observed that normal microbial status can be restored through ASTF intervention.

## Introduction

Inflammation is a protective defensive response against bodily damage involving the participation of a variety of factors and cells [1]. Moderate inflammation supports the health of the body, but excessive inflammation can cause adverse reactions and lead to disease and even death. At present, nonsteroidal and glucocorticoid anti-inflammatory drugs such as amino salicylic acid, dexamethasone etc., are commonly used to treat excessive inflammation, but these drugs have problems such as low effectiveness and negative side effects [2]. As such, natural drugs with few side effects are being sought out as alternatives in treatment. *Acanthopanax senticosus* is a perennial shrub belonging to the genus *Acanthopanax* of the Araliaceae family [3]. Commonly known as Siberian ginseng [4,5], it is one of the commonly used natural Chinese herbal medicines [6,7] and contains a variety of biologically active ingredients, such as saponins, flavonoids and polysaccharides [8]. In previous studies using animal inflammation models, *A. senticosus* and its active ingredients demonstrated anti-inflammatory activity. Wang et al. found that oral administration of *A. senticosus* (3.5 mg/100 g) has a therapeutic effect on sodium taurocholate-induced severe acute pancreatitis in rats, which results from the inhibition of abnormal autophagy activation in pancreatic acinar cells [9]. Fei et al. found that *A. senticosus* attenuated the levels of tumor necrosis factor α (TNF-α) and interleukin (IL)-6 (IL-6) in the lung tissue of a mouse model of acute lung injury by inhibiting the nuclear factor κ B (NF-κB) pathway [10]. Han et al. found that *A. senticosus* polysaccharide may reduce lipopolysaccharide (LPS)-induced sepsis by inhibiting the NF-κB/MLCK pathway [11]. As an important active ingredient, *A. senticosus* flavonoids [6] have anti-inflammatory, antioxidant and antistress effects [12]. Guan et al. found that *A. senticosus* flavone can alleviate the damage caused by myocardial ischemia in rats by improving the function of myocardial calcium channels [13]. However, the effects and mechanisms of *A. senticosus* total flavonoid (ASTF) on LPS-induced intestinal inflammation are not clear at present.

Artificial intraperitoneal injection of LPS results in inflammation of the intestinal mucosa of mice in experiments [14], and LPS-induced intestinal inflammation is often used to develop a model for evaluating the anti-inflammatory effects of drugs [15,16]. LPS induces a mouse model of inflammation and the production of various inflammatory factors and is a key trigger of inflammation [17], which can be recognized by Toll-like receptor 4 (TLR4) [18], and small molecule agonists released by LPS interact with TLR4 to initiate TLR4 dimerization [19], which subsequently aggregates MyD88 by virtue of intracellular structural domains [20], activating the downstream NF-κB p65 subunit. Undergoing phosphorylation to form p-p65 translocated into the nucleus [21], the NF-κB pathway is activated and will mediate the expression of related genes, ultimately triggering the pro-inflammatory mediators and cytokines [22], such as IL-6, IL-1β, TNF-α, prostaglandin E2 (PGE2) and so on [23]. PGE2 is an important mediator involved in inflammatory responses [24], and its synthesis is restricted by COX2.

The intestine is the most vigorous metabolically active organ of the body and represents the largest interface between the environment and the organism in terms of area [25], meaning it is susceptible to external stimuli. Hence, when the intestinal homeostasis environment becomes unbalanced, the intestine is the organ most prone to inflammation. When intestinal inflammation appears, it is often accompanied by disruption of the ecological balance of the microbial flora. However, although intestinal inflammatory diseases (such as IBD) are related to an intestinal microbial imbalance, it is impossible to determine whether the intestinal microbial imbalance is the cause or the result [26,27]. The structure of the intestinal microbes is quite complex, harboring a diverse community of trillions of microorganisms [28]. Among the intestinal microbes of healthy mice, 99% are species of Firmicutes, Bacteroides, Proteobacteria and Actinomycetes, with Firmicutes and Bacteroides accounting for approximately 90% [29]; hence, the intestinal microbial flora is considered balanced based on this profile. Bacteroides and Proteus are Gram-negative bacteria. The unique LPS on the surface of Gram-negative bacteria can cause infection or disease [30], whereas intestine-derived LPS is normal and will not cause intestinal damage within a certain range [31]. Studies have shown that LPS induction can reduce the number and activity of intestinal epithelial cells, inhibit their proliferation and increase apoptosis, ultimately leading to intestinal damage and inflammation [32,33]; in addition, flavonoids can promote the growth of intestinal probiotics, improve the balance of intestinal flora and thereby alleviate the occurrence of inflammation [34]. However, the role and mechanism of ASTFs in mice with intestinal inflammatory disease remain unclear.

An LPS-induced intestinal inflammation model of mice was established to evaluate the anti-inflammatory capability of ASTF *in vivo*, and the anti-inflammatory mechanism of ASTF was elucidated based on analysis of the NF-κB inflammation signal pathway along with the changes in intestinal microbiota, laying the foundation for the clinical application of ASTF.

## Materials and methods

### Materials and instruments

*A. senticosus* decoction samples were purchased from Limin Pharmacy in Liaocheng (Cat# 20200516); LPS (*Escherichia coli* O55:B5) was purchased from Shanghai Aladdin Biochemical Technology Co., Ltd. (Shanghai, China); IL-6, IL-1β, PGE2, and TNF-α ELISA detection kits, SYBR Green qPCR Mix (2×), RIPA lysate, BCA protein concentration determination kit and sodium dodecyl sulfate/polyacrylamide (SDS/PAGE) gel preparation kits were purchased from Shanghai Biyuntian Biotechnology Co., Ltd. (Shanghai, China); MiniBEST Universal RNA Extraction Kit and Prime Script RT Reagent Kit were purchased from TaKaRa Biotechnology Co., Ltd. (Beijing, China). The oligonucleotide primers for mouse: β-actin (β-non-muscle), IL-6, IL-1β, cyclooxygenase-2 (COX-2) and TNF-α were bought from Sangon Biotech Co., Ltd. (Shanghai, China); TLR4 monoclonal antibody (D8L5W,14358), MyD88 monoclonal antibody (D80F5,4283), p65 monoclonal rabbit antibody (D14E12,8242), p-p65 monoclonal rabbit antibody (Ser536,93H1,3033) and β-actin monoclonal antibody (8H10D10,3700) were purchased from Cell Signaling Technology (MA, U.S.A.), Agarose Gel DNA Extraction Kit was purchased from Takara Biotechnology Co., Ltd. (Beijing China).

The oscillating constant temperature metal bath (Shanghai Yiheng Technology Co., Ltd., Shanghai, China) low temperature centrifuge (Hettich Co., Ltd., Shanghai, China), SB-600DTY ultrasonicator (Ningbo Xinzhi Biological Technology Co., Ltd., Zhejiang China), fluorescence spectrophotometer (Thermo Fisher Technology Co., Ltd., Shanghai, China), 1530 microplate reader (Thermo Fisher Technology Co., Ltd., Shanghai, China), horizontal shaker, electrophoresis apparatus, membrane transfer apparatus (Shanghai Biyuntian Biotechnology Co., Ltd., Shanghai, China), CFX Connect Real-Time System (Bio-Rad, Hercules, CA, U.S.A.) and Bio-Rad gel imaging analysis system (Shanghai Fuzhong Biological Science Co., Ltd., Shanghai, China) were sourced as indicated.

### Experimental drugs

ASTF was prepared following the method described in a previous article [35]. In brief, ASTF was extracted with 60% ethanol at a solid–liquid ratio of 1:40, and then sonicated (at 60°C for 65 min) using a SB-600DTY ultrasonicator, before being filtered. After freeze-drying, the conditions for purification of ASTF were determined as a sample pH of 3, 60% ethanol concentration and 3 BV/h flow rate for both adsorption and desorption using 2.5 and 4 volumes of BV, respectively. The contents of seven major flavonoids—protocatechuic acid, isofraxidin, 6′,6′-dimethylpyrano [2′,3′:7,8] flavone, umbelliferone, luteolin, baicalin and baicalein were 11.4892, 1.4791, 0.2432, 1.3348, 1.0353, 5.6938 and 2.4605%, respectively.

### Animals care

A total of 50 specific pathogen free-grade (SPF-grade) healthy Yunnan Kunming mice (5 weeks old, male, weight 17.05 ± 0.51 g) were purchased from Jinan Pengyue Experimental Animal Breeding Co., Ltd, Jinan, China. The mice were raised in an animal room free of specific pathogens in College of Agriculture, Liaocheng University, and were given free access to water and fed a commercial diet. The temperature was controlled at 24 ± 2°C, the relative humidity was 60 ± 5% with a 12-h light–dark cycle. The animals were acclimatized to laboratory conditions for 1 week prior to being subjected to the experiments. All animal experiments were performed in accordance with the Chinese Guidelines of Animal Ethics Committee of Liaocheng University.

### Experimental methods

#### Experimental animal grouping and drug treatment

**Figure 1:**
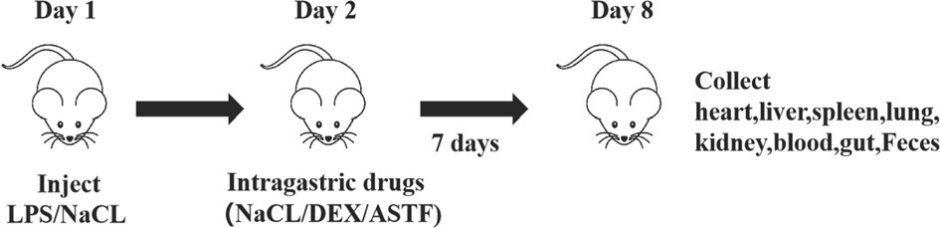
Experimental design

The 50 mice were randomly divided into six groups with eight mice in each group, namely, the control group (control), LPS group (LPS), DEX group (LPS+DEX), ASTF low-dosage group (LPS+L-ASTF), ASTF medium-dosage group (LPS+M-ASTF) and ASTF high-dosage group (LPS+H-ASTF). Except for the control group, all other groups of mice were injected intraperitoneally with 10 mg/kg LPS (0.2 ml) to establish an inflammation model, and the control group was intraperitoneally injected with 0.2 ml of normal saline. After 24 h of injection, the weight of each mouse was measured separately. The control group and the LPS group were intragastrically administered 0.2 ml of normal saline, the LPS+DEX group was intragastrically administered a dose of 1 mg/kg (0.2 ml) and the LPS+L-ASTF, LPS+M-ASTF and LPS+H-ASTF groups—based on the weights of the mice—were administered 200 mg/kg (low dose), 400 mg/kg (medium dose) and 800 mg/kg (high dose) by intragastric administration of 0.2 ml for 7 days. A schematic diagram of the test operation is shown in Figure 1. At the end of the experiment, all mice were killed by cervical dislocation and subject to gross necropsy.

#### Measuring body weight and collecting samples of experimental materials

During the experiment, the stools, coats and mental states of the mice were observed, and the body weights of each group of mice were measured and recorded at a fixed time point every day. After 7 days of gavage, the mice were fasted for 24 h and killed. Prior to killing, the mice were weighed to gain a fasting live weight, and their feces were taken. Blood was collected from the orbital venous plexus, left at room temperature for 1 h and centrifuged for 15 min (1000 rpm); the serum was then collected and stored at −80°C. The mouse heart, lungs, liver, spleen, kidneys and small intestine were taken by dissecting the abdominal cavity after the mice were killed, and the surface debris was washed with normal saline, the weight measured after drying, the organ index calculated and the colon length measured. The isolated ileum, jejunum and duodenum were cut to 1-cm lengths and fixed in 4% paraformaldehyde for HE staining and PAS pathological sectioning. The remaining small intestine tissue was made into a homogenate with a 10% concentration with 0.9% sodium chloride and centrifuged for 15 min (700 rpm), and the supernatant was frozen and stored at −80°C.

#### Hematoxylin–Eosin staining

Paraformaldehyde-fixed tissues were embedded, sliced and HE stained, and histopathological changes were observed under a microscope. Three fields of view as observed under an optical microscope were used for comparison of each group. ImageJ software was used to select and measure six relatively complete villi and six crypts for each tissue section to compare the villus height (VH):crypt depth (CD).

#### Periodic acid–Schiff stain

Paraformaldehyde-fixed tissues were embedded, sliced and PAS stained, and the goblet cells (GCs) in the tissues were observed under a microscope. Three fields of view were as observed under an optical microscope were used for comparison of each group, and the number of GCs was counted.

#### Enzyme-linked immunosorbent assay

The serum of each mouse in each group was collected, and the contents of IL-1β, IL-6, PGE2 and TNF-α in the mouse serum were determined according to the instructions of the enzyme-linked immunosorbent assay (ELISA) test kit.

#### Detection of relative mRNA expression by real-time PCR

Total RNA was extracted from mice small intestine tissue using reagent and transcribed into cDNA according to the manufacturer’s instructions. RT-PCR was performed on a real-time system spiked with SYBR Green to detect mRNA levels of IL-1β, IL-6, COX-2 and TNF-α. RT-PCR was performed in a 20-µl system under the following program: pre-denaturation 95°C for 2 min, followed by 40 cycles of 95°C for 15 s and 60°C for 30 s. Quantification was performed using *β-actin* as an internal reference gene to normalize gene expression of target genes, and primers specific for each gene are listed in Table 1. All experiments were performed in three biological replicates and three technical replicates. The relative expression of mRNA level was calculated with the 2^−ΔΔ*C*_t_^ method.

**Table 1:** Sequence of primers for RT-PCR

Gene	Primer sequences (5′–3′)		Fragment size (bp)
*β-actin*	Sense	CCTTCTCTCTCTCTCCCTCTTT	118
	Antisense	CTCAGTGTCTGCCATCTTCTAC	
*IL-1β*	Sense	AGTTGACGGACCCCAAAAG	80
	Antisense	TTTGAAGCTGGATGCTCTCAT	
*IL-6*	Sense	CCACTCACCTCTTCAGAACG	150
	Antisense	CATCTTTGGAAGGTTCAGGTTG	
*COX-2*	Sense	GCTCAGCCAGGCAGCAAAT	176
	Antisense	TTGGGGTGGGCTTCAGCAGT	
*TNF-α*	Sense	GCTCTTCTGTCTACTGAACTTCGG	110
	Antisense	ATGATCTGAGTGTGAGGGTCTGG	

#### Western blot analysis

The frozen intestinal tissue was taken and whole tissue protein was extracted to prepare the Western blot lysate, with the whole process being carried out on ice. BCA protein analysis method was used to detect the protein concentration. We mixed the protein sample with 5× buffer in the appropriate proportion, heated it at a high temperature to denature it and then stored it at −20°C. A 30-μg aliquot of protein sample was loaded on a 10% concentrated SDS/PSGE gel (60 V) and 10% separation gel (120 V), and transferred it to a PVDF membrane under 300 mA current. The membrane was shaken during blocking with 5% skimmed milk for 3 h and incubated with the appropriate diluted primary antibody (dilution ratio: β-actin 1:10000; TLR-4 1:1000; MyD88 1:1000; p65 1:1000; p-p65 1:1000) with incubation overnight at 4°C. The membrane was then washed with TBST solution three times, for 15 min each time, then incubated with the diluted secondary antibody for 1.5 h with shaking at room temperature before washing with TBST for 15 min for a total of three times. We utilized ECL chemical reagents to develop the color and analyze the relative protein abundance.

#### Intestinal microbes

Total genome DNA from samples was extracted using CTAB/SDS method. The bacterial primer pair 338F (5′-ACTCCTACGGGAGGCAGCA-3′) and 806R (5′-GGACTACNNGGG TATCTAAT-3′) were used to amplify the V3–V4 hypervariable regions of the bacterial 16S rRNA gene [36], the amplified products were retrieved and purified by Agarose Gel DNA Extraction Kit and mixed in equimolar concentrations. Then, the purified amplicons were quantified using a fluorescence spectrophotometer (Thermo Scientific, MA, U.S.A.) [37]. Sequencing libraries were generated using NEB Next Ultra DNA Library Prep Kit for Illumina (NEB, U.S.A.) following manufacturer’s recommendations and index codes were added. At last, the library was sequenced on an Illumina MiSeq platform and 250 bp/300 bp paired-end reads were generated. Data were analyzed using a software platform (tutools, https://www.cloudtutu.com/#/login).

### Statistical analysis

The data were analyzed using the SPSS 21.0 statistical software, and the experimental results were all expressed as the means ± standard deviations. All the experiments were repeated three times. The statistical processing of the data used one-way analysis of variance (ANOVA), and the difference was tested as the means between multiple groups. Figures were made using the Origin 2021 pro.

## Results and discussion

### Effect of ASTF on body weight of LPS-induced mice

**Figure 2:**
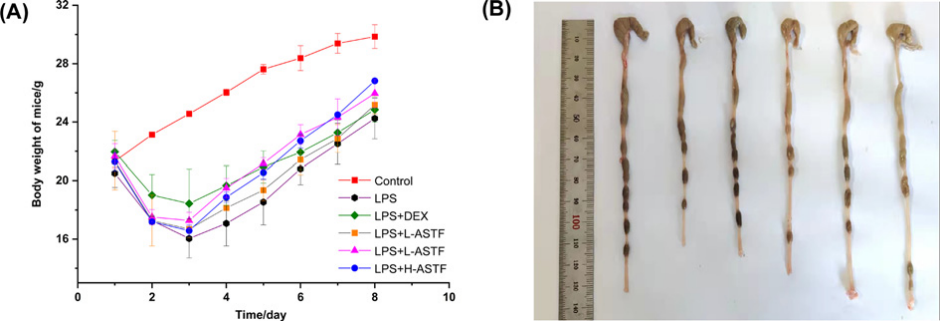
The body weight and colon length of mice in six groups (**A**) The body weight of each group. (**B**) Colon length (mm) of each group.

The weight changes of mice in each group are shown in Figure 2A. Prior to intraperitoneal LPS injection, there was no significant difference in the weight of mice in each group (*P*>0.05). After 24 h of LPS treatment, the weight of mice in the blank control group increased normally, whereas the weight in the other groups was reduced. From the fourth day of the experiment, these weights then began to increase gradually, but were not able to reach the values recorded for the weights of the normal group of mice as observed up to the end of experiment. Among them, the final weight of the LPS+H-ASTF group showed the highest increase, by 126.03% of the initial weight. The body weight of the mice in the LPS group also increased slightly after 7 days, to 113.15% of the initial body weight. At the end of the experiment, the mice in the LPS group still had the lowest body weight.

### Effect of ASTF on mental state, coat and diarrhea of LPS-induced mice

The mice in the normal group had normal coats, activity levels, food intakes and defecation habits. The mice treated with LPS were observed after 2 h, and it was found that the mental state of the mice was slightly poorer, but there was no diarrhea. After 6 h of LPS treatment, the mice began to show signs of illness, with messy coats and diarrhea (yellow loose stools) of varying degrees displayed by approximately two to four mice in each group, except the control group. Throughout the experiment, the mice in the LPS group showed varying degrees of diarrhea, blood in the stool, loss of appetite and messy coats, while the mice in the LPS+DEX group and the *A. senticosus* group performed well and gradually regained their vitality under the drug treatment. Their stools were normal and the amount of blood in the stool was reduced.

### Effect of ASTF on organ index and colon length in LPS-induced mice

Generally speaking, the increase in the organ index value in mice indicates the appearance of pathological phenomena such as hyperemia and hypertrophy in the organs, while a decrease indicates the occurrence of organ atrophy and the decline in physiological function. As shown in Table 2, compared with the control group, the value for spleen index of the mice in the LPS group increased significantly (*P*<0.05), and those of the heart, liver, lungs and kidney also increased, but there was no significant difference (*P*>0.05). Compared with the LPS model group, the LPS+DEX group had reduced index values for each organ; that for the lung index was significantly reduced, but the rest were not significantly different. Dose-dependent reductions in the index values for various organs were observed in the *A. senticosus* flavonoids group, but the changes were not significant. As shown in Figure 2B, compared with the blank control group, the length of the mouse colon in the LPS group was significantly shortened. Meanwhile, the colon lengths of the mice in LPS+DEX group increased, and the colon lengths of the mice in the* A. senticosus* flavonoids group increased in a dose-dependent manner.

**Table 2:** Organ index in mice of six groups

Group	Heart index (%)	Liver index (%)	Spleen index (%)	Lung index (%)	Kidney index (%)
Control	0.63 ± 0.04^1^	5.17 ± 0.39^1^	0.45 ± 0.08^2^	0.72 ± 0.07^1^	1.39 ± 0.11^1^
LPS	0.67 ± 0.04^1^	5.55 ± 0.36^1^	0.73 ± 0.09^1^	0.76 ± 0.09^1^	1.55 ± 0.08^1^
LPS+DEX	0.62 ± 0.07^1^	4.83 ± 0.43^1^	0.40 ± 0.15^2^	0.71 ± 0.01^1^	1.37 ± 0.23^1^
LPS+L-ASTF	0.64 ± 0.12^1^	5.11 ± 0.51^1^	0.66 ± 0.1^1,2^	0.75 ± 0.06^1^	1.40 ± 0.09^1^
LPS+M-ASTF	0.63 ± 0.07^1^	5.07 ± 0.43^1^	0.64 ± 0.08^1,2^	0.73 ± 0.05^1^	1.38 ± 0.11^1^
LPS+H-ASTF	0.60 ± 0.03^1^	4.82 ± 0.34^1^	0.58 ± 0.08^1,2^	0.71 ± 0.01^1^	1.33 ± 0.11^1^

Data are presented as mean ± SD (*n*=8).

Organ index (%) = wet organ weight/body weight × 100%.

In the same column, values with different superscript numbers (1,2) mean significantly different (*P*<0.05), while those with the same superscript numbers mean not significantly different (*P*>0.05).

### Effects of ASTF on small intestine of LPS-induced mice

**Figure 3:**
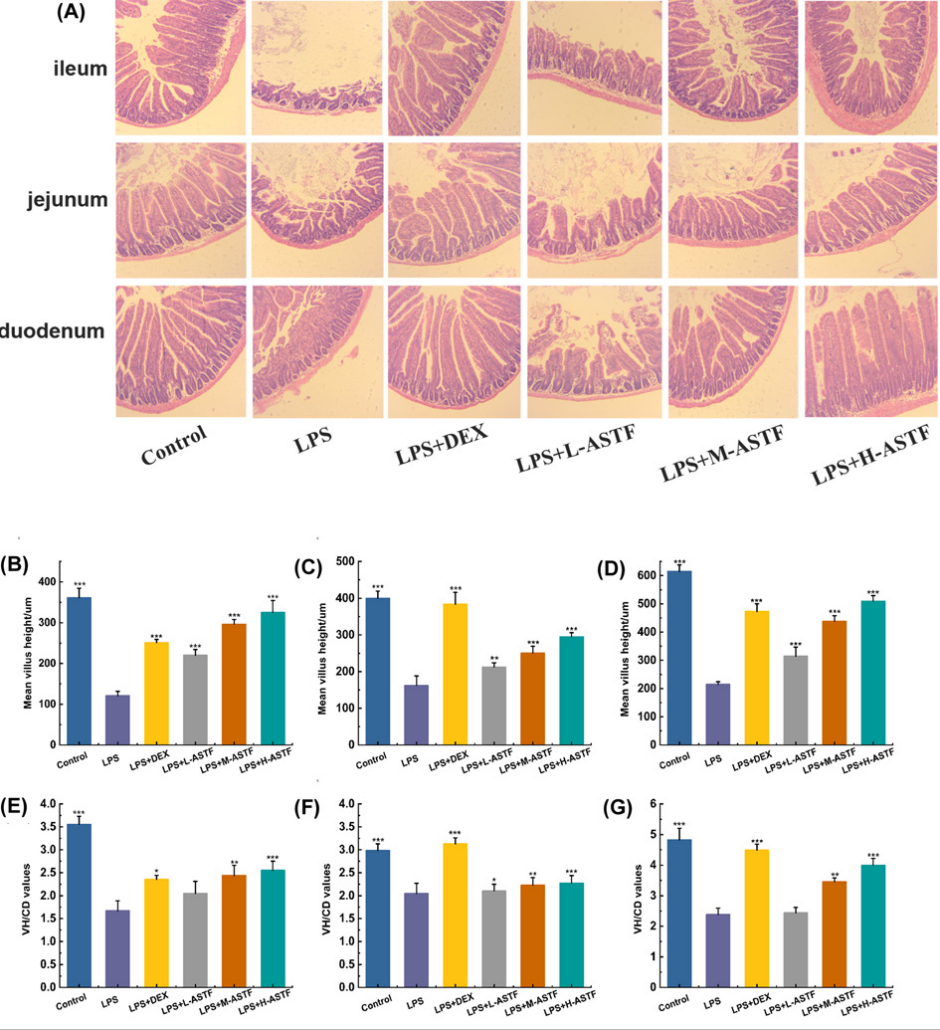
Effects of ASTF on morphology of ileum, jejunum and duodenum in LPS-induced mice (**A**) HE staining of ileum, jejunum and duodenum. (**B–D**) VH of ileum, jejunum and duodenum in each group. (**E–G**) Ratio of VH/CD of ileum, jejunum and duodenum in each group. Compared with the LPS group, *P<0.05, **P<0.01, ***P<0.001.

As seen in Figure 3A, the intestinal wall of the small intestine tissue (ileum, jejunum, duodenum) of the control group of mice are intact, the structures are clear, the villi are neatly arranged, there is an obvious crypt structure and no evidence of inflammatory cell infiltration. By contrast, the intestinal wall of the mice in the LPS model group appears destroyed and necrotic, the small intestine villi have fallen off, the crypt structure has completely disappeared and there are a large number of inflammatory cells. In the LPS+DEX and LPS+H-ASTF groups, the small intestine tissue of the mice has basically returned to normal and the intestinal wall appears healthy. The crypts appear normal and the inflammatory cells have disappeared, which demonstrates that the treatment with ASTF was beneficial. Only a slight therapeutic effect was observed in the LPS+L-ASTF group had, with better effects in the LPS+M-ASTF group, though some inflammatory cells could still be observed. In general, ASTF was found to have a dose-dependent, beneficial therapeutic effect on intestinal disease in the treated mice.

As shown in Figure 3B–G, the VH and CD of the small intestine (ileum, jejunum, duodenum) represent, to a certain extent, the shape of the small intestine and its digestion and absorption capacity. The shortening of villi and the reduction in the VH/CD ratio all indicate the presence of inflammation in the intestinal mucosa of the small intestine. Compared with the control group, the VH of the ileum, jejunum and duodenum in the LPS model group were 33.44, 40.57 and 34.98% of those in the normal group, respectively, and the VH/CD ratio was 47.02, 59.34 and 49.42% of the normal group. In all cases, the findings were considered significantly different (*P*<0.001), indicating that the intraperitoneal injection of mice with LPS was successful in inducing intestinal inflammation. Compared with the LPS model group, in the LPS+L-ASTF group of mice, the L-ASTF treatment only significantly increased the villi of the ileum (*P*<0.001) and not the jejunum or duodenum (*P*<0.01), though the VH:CD of the jejunum intestinal segment was significantly increased (*P*<0.05). The VH of the intestinal segment of the small intestine of the LPS+M-ASTF group was extremely significantly increased (*P*<0.001), and the VH:CD values significantly increased (*P*<0.01). The VH of the whole small intestine in the LPS+H-ASTF group was significantly increased (*P*<0.001), and the ratio of VH:CD was extremely significantly increased (*P*<0.001). In addition, the LPS+H-ASTF group and LPS+DEX group have better performance in terms of various indicators, and the effect was greater in the LPS+ASTF group. Overall, the observations demonstrate that ASTF can improve the intestinal morphology of LPS-induced mice in a dose-dependent manner.

### Periodic acid–Schiff stain

**Figure 4:**
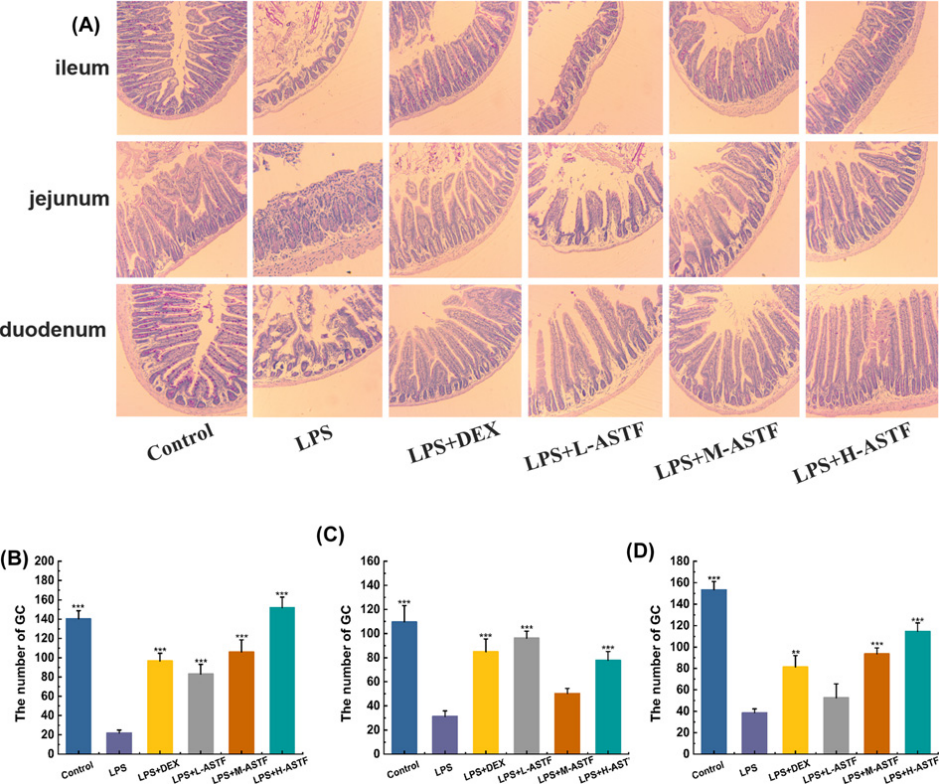
Effect of ASTF on GCs of ileum, jejunum and duodenum in mice induced by LPS (**A**) PAS staining of each group. (**B**) Number of GC in ileum. (**C**) Number of GC in jejunum. (**D**)Number of GC in duodenum. Compared with the LPS group, **P<0.01, ***P<0.001.

GCs distributed in the digestive tract of mammals are goblet-shaped, highly polarized columnar epithelial cells that mainly secrete mucin MUC2 [38], a key component of the mucosal barrier [39]. The mucus layer is in the intestinal contents and intestine epithelial cells; it plays a role in lubrication and buffer barriers, and GCs directly determine the mucus quality [40]. With PAS staining, Schiff reagent can stain GCs red or purple, showing a strong positive reaction. As shown in Figure 4, compared with the control group, the GCs in the ileum, jejunum and duodenum of the LPS group were significantly reduced (*P*<0.001), and ASTF can increase the number of GCs. The effects of ASTF in the ileum and duodenum are dose-dependent, but in the jejunum, L-ASTF has the greatest effect on GCs.

### Detecting the contents of IL-1β, IL-6, PGE2 and TNF-α in mouse serum by ELISA

**Figure 5:**
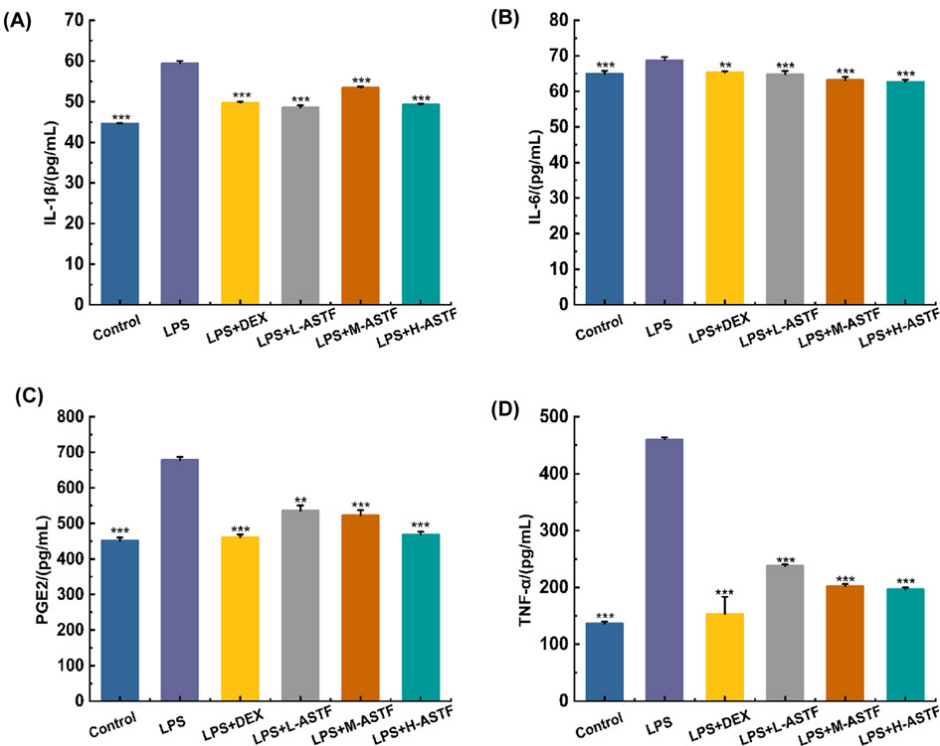
Effects of ASTF on IL-1β, IL-6, PGE2and TNF-α in serum of mice induced by LPS (**A**) The changes of IL-1β content in each group. (**B**) The changes of IL-6 content in each group. (**C**) The changes of PGE2 content in each group. (**D**) The changes of TNF-α content in each group. Compared with the LPS group, **P<0.01, ***P<0.001.

To study whether ASTF can reduce the production of inflammatory factors in mice stimulated by LPS, the levels of IL-1β, IL-6, PGE2 and TNF-α in mouse serum were determined using ELISA, and the anti-inflammatory activity of ASTF was studied. As shown in Figure 5, the levels of IL-1β, IL-6, PGE2 and TNF-α in the LPS model group were significantly higher than those in the control group (*P*<0.001), indicating that the mice had severe inflammation after intraperitoneal injection of LPS. The serum levels of IL-1β, IL-6, PGE2 and TNF-α in the LPS+DEX group were significantly lower than those in the LPS model group (*P*<0.001), and compared with the control group, the contents of IL-6, PGE2 and TNF-α were not significantly different, indicating that DEX has a good therapeutic effect. Compared with the LPS model group, ASTF significantly reduced the contents of IL-1β, IL-6, PGE2 and TNF-α in each dosage group (*P*<0.001), and except for the content of IL-1β, all of these effects were dose-dependent. The LPS+H-ASTF group had significantly reduced contents of IL-1β, IL-6, PGE2 and TNF-α (*P*<0.001), and these contents are close to Control group. The results show that LPS can induce inflammation in mice and that ASTF can significantly reduce the levels of IL-1β, IL-6, PGE2 and TNF-α in mouse serum and alleviate the inflammatory response in mice.

### Detecting the relative mRNA expression of IL-1β, IL-6, PGE2 and TNF-α in mouse serum by RT-PCR

**Figure 6:**
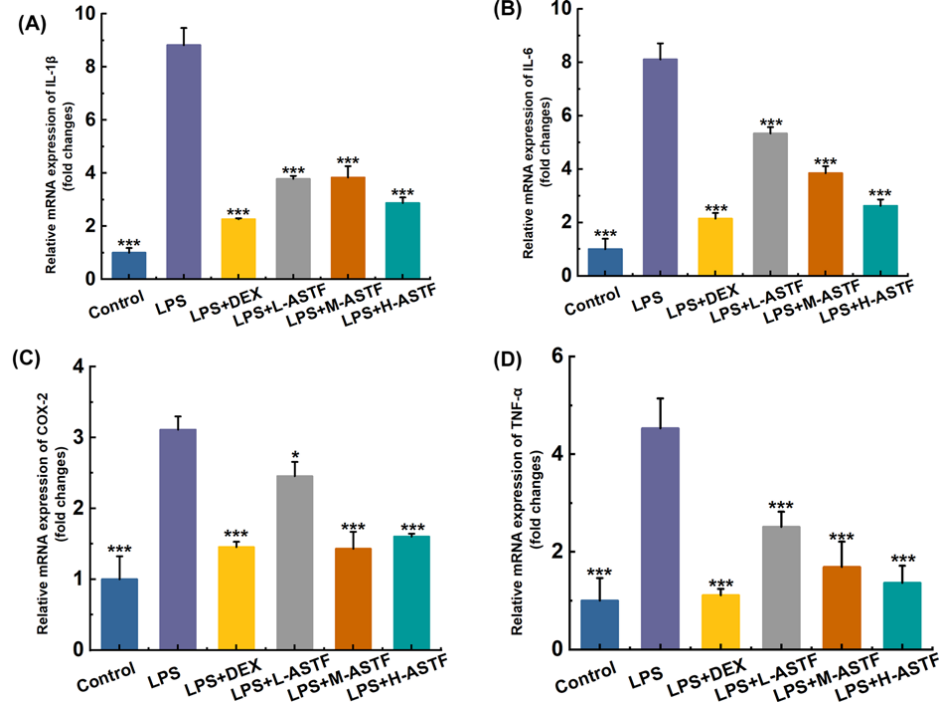
Effects of ASTF on relative mRNA expression of IL-1β, IL-6, COX-2 and TNF-α in serum of mice induced by LPS (**A**) The expression of IL-1β mRNA in the Control group was taken as 1, and the expression in the remaining groups was expressed as a multiple of the Control group. (**B**) The expression of IL-6 mRNA in the Control group was taken as 1, and the expression in the remaining groups was expressed as a multiple of the Control group. (**C**) The expression of COX-2 mRNA in the Control group was taken as 1, and the expression in the remaining groups was expressed as a multiple of the Control group. (**D**) The expression of TNF-α mRNA in the Control group was taken as 1, and the expression in the remaining groups was expressed as a multiple of the Control group. Compared with the LPS group, *P<0.05, ***P<0.001.

To investigate whether ASTF can inhibit the mRNA expression levels of inflammatory factors and mediators in LPS-stimulated mice, RT-PCR was used to measure the relative mRNA expression of IL-1β, IL-6, COX-2 and TNF-α in mouse serum, and the anti-inflammatory activity of ASTF was investigated. As shown in Figure 6, the mRNA expression levels of IL-1β, IL-6, COX-2 and TNF-α in the small intestinal tissue of mice in the LPS model group were significantly higher than those in the control group (*P*<0.001), indicating that intraperitoneal injection mice of after LPS exhibited severe inflammation. The mRNA expression levels of IL-1β, IL-6, COX-2, and TNF-α in the LPS+DEX group were significantly lower than those in the LPS model group (*P*<0.001), and DEX had a good therapeutic effect. Compared with the LPS model group, LPS+L-ASTF can significantly reduce the mRNA expression of COX-2 (*P*<0.05), and the ASTF of other dose significantly reduced IL-1β, IL-6, COX-2, TNF-α mRNA expression level (*P*<0.001), and the inhibitory effect of ASTF on IL-6 and TNF-α mRNA expression was dose-dependent. The results show that LPS can induce inflammation in mice, and ASTF can significantly reduce the relative mRNA expression levels of IL-1β, IL-6, COX-2 and TNF-α in the small intestinal tissue of mice, and reduce the degree of inflammation in mice.

### Western blot assay

**Figure 7:**
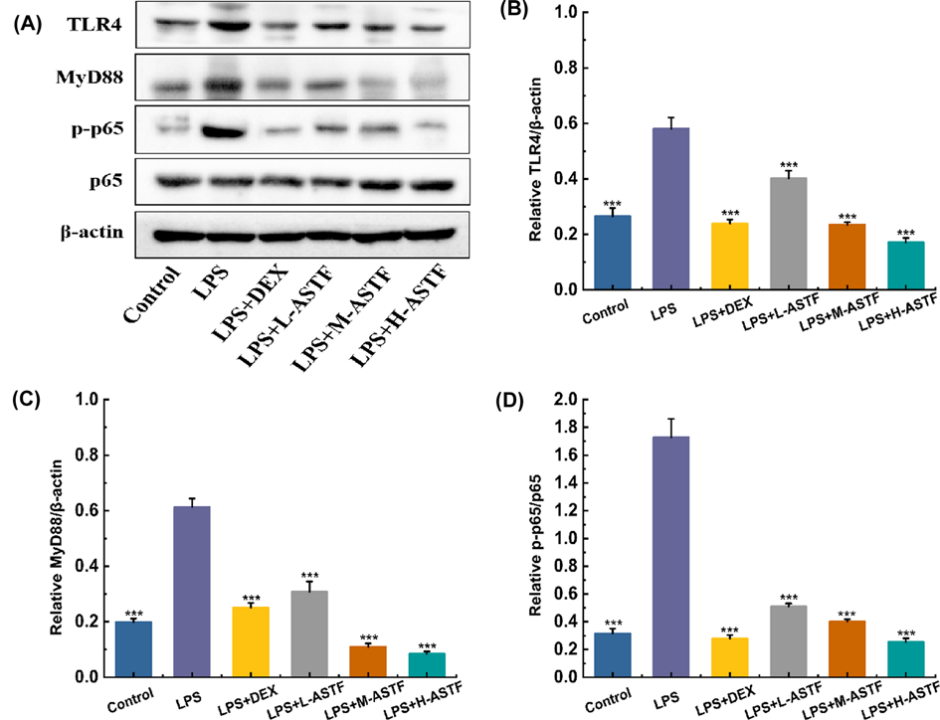
Effects of ASTF on expression of proteins in the NF-κB pathway (**A**) Western blot images. (**B**) Relative protein expression of TLR4. (**C**) Relative protein expression of MyD88. (**D**) Relative protein expression of NF-κB p-p65. Compared with the LPS group, ***P<0.001.

To further explore the mechanism of the therapeutic effect of ASTF on intestinal inflammation in mice, the effect on the expression of related proteins in the NF-κB pathway was studied. After the protein in the small intestinal tissue was extracted using a reagent test kit, a Western blot was carried out to detect the expression levels of the TLR-4, MyD88, NF-κBp65 and NF-κBp-p65 proteins. The gray value of all bands was analyzed using ImageJ, and normalized according to the corresponding internal control protein amount, with the results shown in Figure 7.

It can be seen from Figure 7B,C that the protein content of TLR4 and MyD88 in the LPS model group is increased compared with the control group, indicating that after LPS stimulation, there is binding of LPS to the TLR4 receptor, which activates and increases the expression of the MyD88 protein, followed by activation of the NF-κB pathway. Compared with the LPS model group, the contents of TLR4 protein in the LPS+DEX group and the ASTF group were significantly reduced (*P*<0.001). As shown in Figure 7D, the expression level of p-p65 protein in the LPS model group was significantly higher than that in the blank group (*P*<0.001), indicating that the phosphorylation level increased. After phosphorylation, the p65 protein translocates to the nucleus, causing inflammation. The LPS+DEX and ASTF groups had reduced levels of p65 phosphorylation, and the inhibitory effect of ASTF was dose-dependent. The LPS+M-ASTF group and the LPS+H-ASTF group had significantly reduced levels of p-p65 protein (*P*<0.001). In summary, the results indicate that ASTF can reduce intestinal inflammation in mice and therefore be used in treatment. This may be because ASTF can regulate the expression level of relevant proteins in the NF-κB pathway. Upon inhibiting the binding of LPS to the TLR4 receptor, the expression of the MyD88 protein is reduced, affecting the phosphorylation level of p65 protein, which means that p-p65 cannot translocate to the nucleus to participate in orchestrating anti-inflammatory effects.

### Effect of ASTF on intestinal flora of mice

#### Operational taxonomic unit level

**Figure 8:**
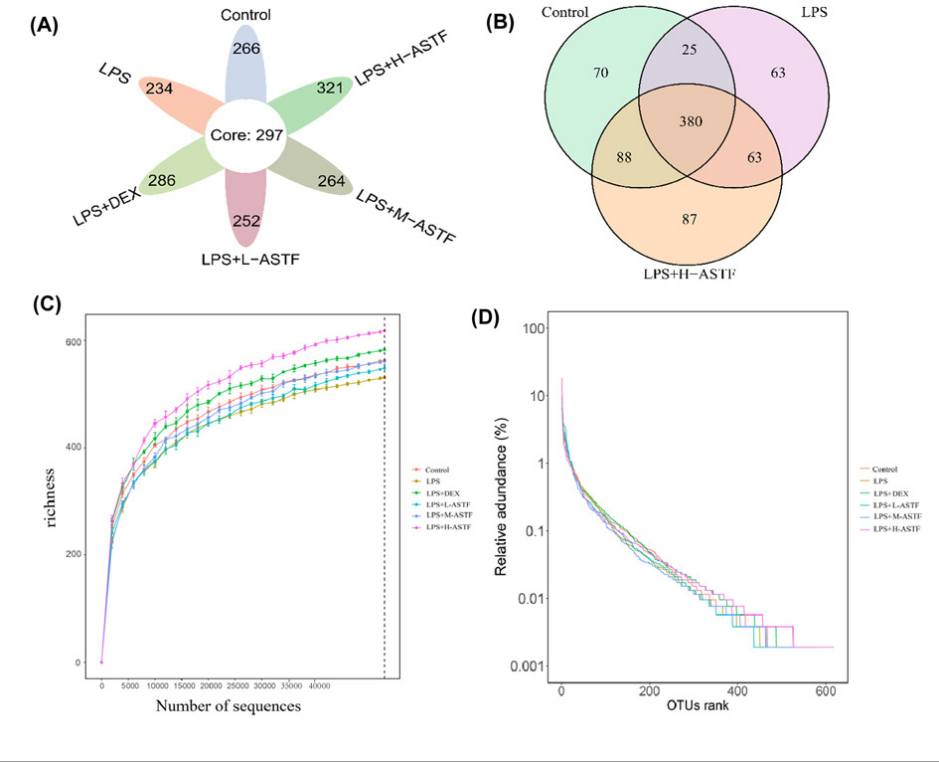
Number of bacterial OTUs in mice feces in six groups (**A**) Petal plot, where the core region indicating the number of OTUs among between groups. (**B**) Venn diagram, where areas of overlap indicate the numbers of OTUs shared among the overlapping groups. (**C**) Rarefaction curves showing the number of OTUs recovered at different sequencing depths. (**D**) Rank abundance curves showing sample diversity.

A total of 1444598 16S rRNA gene sequences were identified in all mouse fecal samples, and all sequences were classified at the 97% similarity level based on operational taxonomic unit (OTU) and according to subsequent bioinformatics. In six groups of mouse stool samples—namely, the control, LPS, LPS+DEX, LPS+L-ASTF, LPS+M-ASTF and LPS+H-ASTF—a total of 1038 OTUs, 7 phyla, 11 classes, 12 orders, 19 families and 47 genera were identified. To more clearly show the number of OTUs in the intestines of mice in different groups, a petal map was made as shown in Figure 8A. We identified 297 core OTUs in all samples, 266 OTUs unique to the control group, 234 OTUs unique to the LPS group, 286 OTUs unique to the LPS+DEX group, 252 OTUs unique to the LPS+L-ASTF group, 264 OTUs unique to the LPS+M-ASTF group and 321 OTUs unique to the LPS+H-ASTF group. To compare the existence of OTUs in the control, LPS and LPS+H-ASTF groups, a Venn diagram (Figure 8B) was constructed. There were 405 identical OTUs between the control and LPS groups, and 468 identical OTUs between the LPS and LPS+H-ASTF groups. Compared with the LPS model group, the OTU types in mice of the LPS+H-ASTF group were closer to those of mice in the control group, which demonstrates the effective restoration of OTU type in the intestines based on H-ASTF treatment. As shown in Figure 8C, by drawing a sparse curve of each group at the OTU level, it was found that all curves tended to stabilize, indicating that a large number of microorganisms present in the samples were captured at this sequencing level, and that the result is credible. A rank abundance curve is given in Figure 8D. In the horizontal direction, the curve width reflects the abundance of the species; the LPS+H-ASTF group has the widest range, followed by the LPS+DEX group and the control group, indicating that the order of increasing species abundance is LPS+H-ASTF, LPS+DEX, control group.

#### α analysis

**Figure 9:**
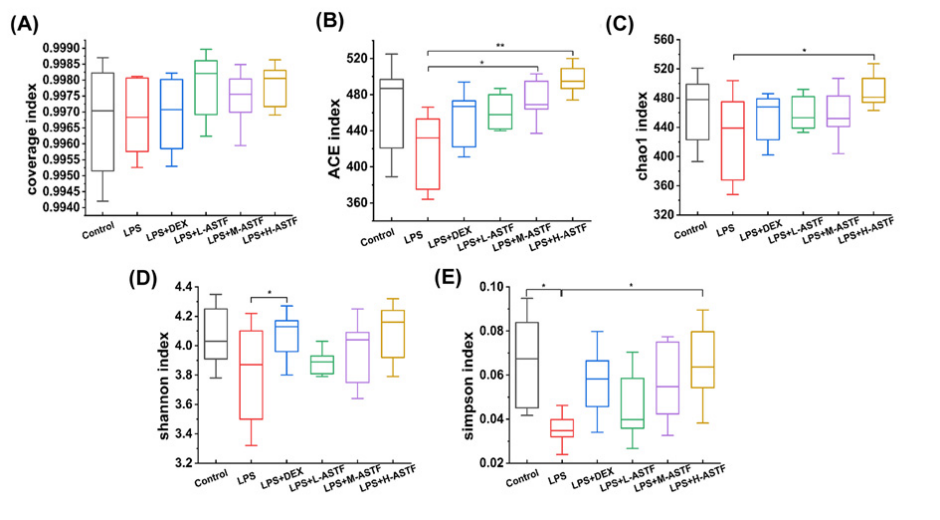
α-diversity index statistics of the fecal microbiota in mice of six groups (**A**) Coverage index. (**B**) ACE index. (**C**) Chao1 index. (**D**) Shannon index. (**E**) Simpson index. Compared with the LPS group, *P<0.05, **P<0.01.

In further analysis of the abundance and diversity of microorganisms in all samples, the ACE, Chao1, Shannon and Simpson indexes, along with the coverage, were determined for the six groups. As shown in Figure 9A, the coverage of the six groups was higher than 0.99 in all cases, indicating that more than 99% of the flora in all samples were captured. For the two indexes of ACE (Figure 9B) and Chao1 (Figure 9C), the values of all other groups were higher compared with the LPS group. In addition, the ACE values of the LPS+M-ASTF group and LPS+H-ASTF group were significantly higher than that of the LPS group (*P*<0.05, *P*<0.01). The Chao1 value of the LPS+H-ASTF group was significantly higher than that of the LPS group (*P*<0.05), indicating that the LPS group had the lowest species richness, and that LPS+H-ASTF can significantly improve the species richness of the small intestine. For the Shannon and Simpson indexes, the LPS group had the lowest value. As shown in Figure 9D, the Shannon value of the LPS+DEX group was significantly higher than that of the LPS group (*P*<0.05), and the Simpson values of the control and LPS+H-ASTF groups were significantly higher than those of the LPS group (*P*<0.05) can be seen in Figure 9E, indicating that the species and richness diversity of the mouse small intestine are reduced after LPS stimulation, and that ASTF can increase the richness diversity of the mouse intestinal flora.

#### β analysis

**Figure 10:**
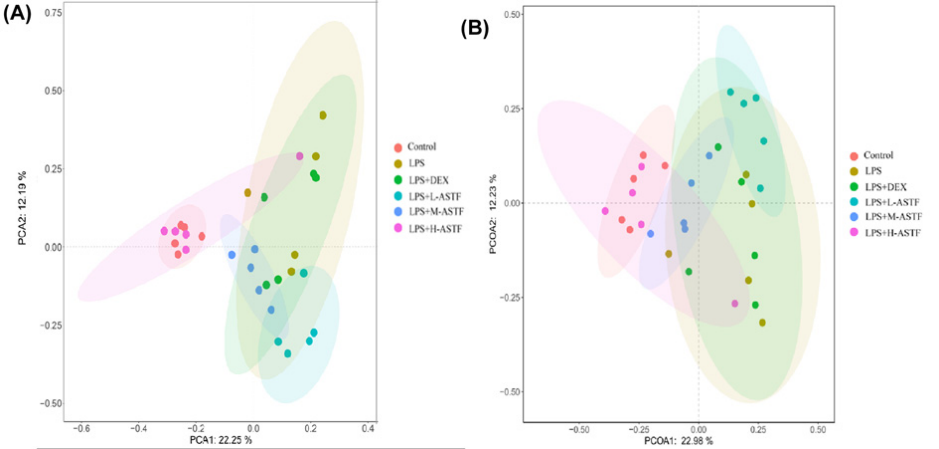
β-diversity analysis of fecal microorganisms in mice of six groups based on OTU levels (**A**) PCA based on OTU level. (**B**) PCoA based on OTU-level. Each point represents a sample and the distance between points is used to map the size of the difference in the structural composition of the gut flora between the two samples.

For β-diversity, as shown in Figure 10, principal component analysis (PCA) and principal coordinate analysis (PCoA) analysis were used to compare the similarity of the microbial community structures in different groups. Figure 10A shows that pca1 and pca2 explained 22.25 and 12.19%, respectively, of the differences in sample composition. The explanations of pcoa1 and pcoa2 for the difference in sample composition would have been 22.98 and 12.23% are shown in Figure 10B, respectively, except that the samples in the LPS group were scattered, while the samples in the other groups were more clustered. The control group and the LPS group had the farthest coordinate distances, indicating that LPS disturbs the microbial population structure of the mouse intestine and that the microbial structure compositions between the two are quite different. The samples of the LPS+H-ASTF group were very similar to those of the control group, indicating that H-ASTF treatment can restore the gut microbial population disrupted by LPS.

#### ASTF regulates taxonomic composition of intestinal microbiota

**Figure 11:**
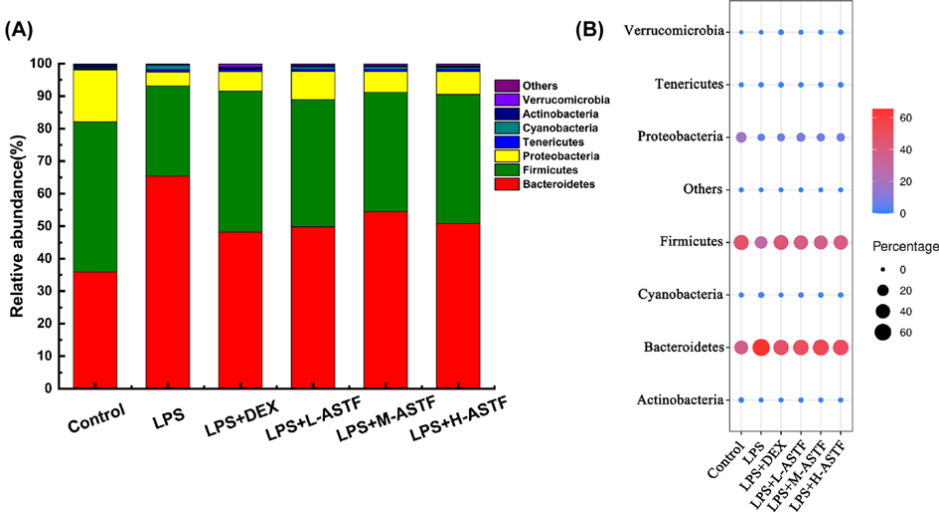
Microbial community composition in mouse feces at phylum levels (**A**) Barplot chart. (**B**) Bubble chart; the size of the black dots and the different colors indicate changes in composition. The figure reflects the representation of the seven main microorganisms in the six groups at the phylum level.

Composition structure analysis of each of the bacterial groups at the phylum level is shown in Figure 11. The types of components were similar in the intestinal flora of each group of samples, but the proportions differ. The dominant phylum was Bacteroides, Firmicutes and Proteobacteria. Other important bacterial phyla are Tenericutes, Cyanobacteria, Actinobacteria, and Verrucomicrobia. Firmicutes regulate the inflammatory response by promoting the secretion of anti-inflammatory mediators, and the proportions of Proteobacteria and Bacteroides are positively correlated with the degree of inflammation [41]. As shown in Figure 11A, the proportions of Firmicutes in the six groups of control, LPS, LPS+DEX, LPS+L-ASTF, LPS+M-ASTF and LPS+H-ASTF were 46.198, 27.754, 43.325, 39.092, 36.723 and 49.714%, while the Bacteroides accounted for 35.897, 65.38, 48.175, 49.818, 54.458 and 50.817% and the Proteobacteria accounted for 15.999, 4.264, 5.989, 8.751, 6.335 and 7.025%, respectively. As shown in Figure 11B, compared with the control group, the proportion of Firmicutes in the stool samples of the LPS group decreased, and the proportion of Bacteroides increased, while ASTF can change the composition of the microbial population such that the disrupted population returns.

**Figure 12:**
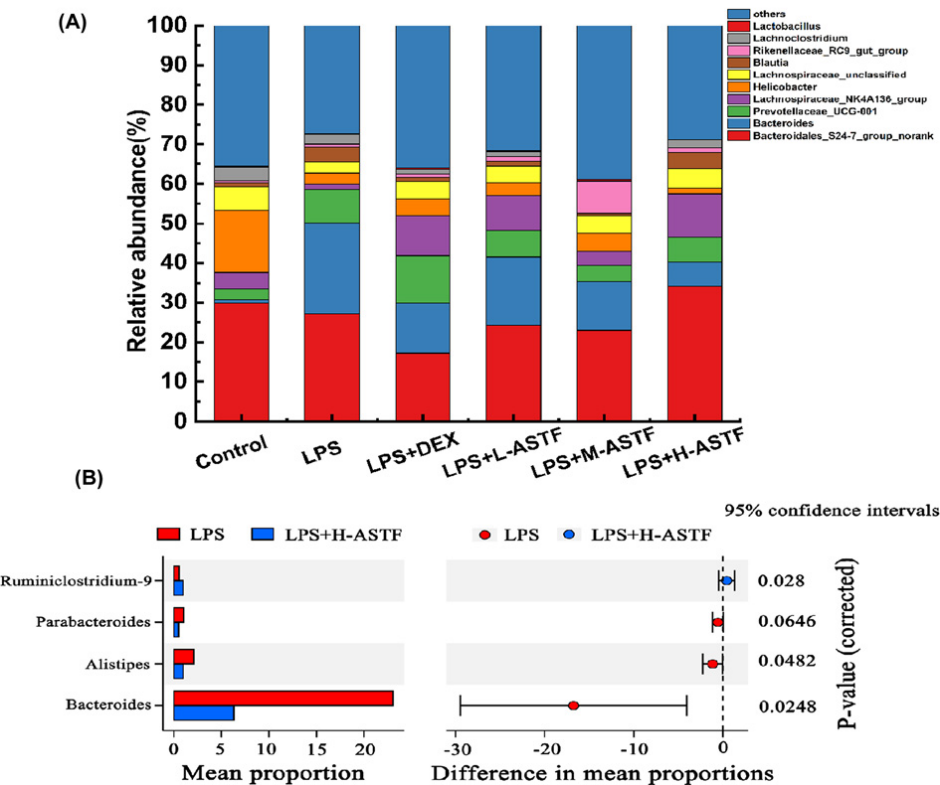
Microbial community composition in mouse feces at genus levels (**A**) Barplot chart. (**B**) Stamp variance analysis between LPS group and LPS+H-ASTF group. *P*-value indicates the level of significance of the four bacteria in the two groups.

The species abundance in the control, LPS, LPS+DEX, LPS+L-ASTF, LPS+M-ASTF and LPS+H-ASTF groups at the genus level is shown in Figure 12A. A total of 47 genera were identified, among which the relatively highly represented genera included *Bacteroidales_S24-7_group_norank*, *Bacteroides*, *Prevotellaceae_UCG-001* and *Lachnospiraceae_NK4A136_group*. The proportions of *Bacteroidales_S24-7_group_norank* in each group were 29.93, 27.12, 17.16, 24.31, 22.91 and 33.99%, respectively. The proportions of *Bacteroides* in each group were 0.86, 23.07, 12.79, 17.22, 12.41 and 6.30%, respectively. The proportion of *Bacteroides* in the intestinal microbes of mice in the LPS group was significantly increased, and ASTF intervention reduced the proportion of *Bacteroides*. In addition, the proportion of *Lactobacillus* in the intestinal microbes of the LPS group of mice was significantly decreased, while the proportion of *Bacteroides* increased after ASTF intervention. As can be seen from Figure 12B, at the genus level, the proportions of *Bacteroides* and *Alistipes* of the LPS+H-ASTF group were significantly reduced (*P*<0.05) compared with the LPS group, while *Parabacteroides* were decreased and the proportion of *Ruminiclostridium_9* increased.

## Conclusion

To our best knowledge, this is the first report of the anti-inflammatory effects of ASTF on LPS-induced intestinal inflammation in mice. ASTF exerted an anti-inflammatory effect by inhibiting intestine injury, mitigating inflammation, preserving the integrity of the intestinal barrier and regulating gut microbiota homeostasis. In our work, the relevant pharmacological activity was determined, as well as the anti-inflammatory mechanism, which may occur through the TLR4/NF-κB signaling pathway of ASTF. These findings provide evidence that ASTF has significant anti-inflammatory properties and could serve as a potential drug for use in the treatment of inflammatory diseases.

## Supplementary Material

Supplementary Data

## Data Availability

The data used to support the result of the present study can be obtained from the corresponding authors.

## Abbreviations

ASTF: *Acanthopanax senticosus* total flavonoid
BV: Bed volume
CD: crypt depth
COX-2: cyclooxygenase-2
ELISA: enzyme-linked immunosorbent assay
GC: goblet cell
HE: Hematoxylin–eosin
IBD: Inflammatory bowel disease
IL: interleukin
LPS: lipopolysaccharide
MLCK: Myosin light chain kinase
NF-κB: nuclear factor κ B
OTU: operational taxonomic unit
PAS: Periodic acid–Schiff
PGE2: prostaglandin E2
TLR4: Toll-like receptor 4
TNF-α: tumor necrosis factor α
VH: villus height
