# Supplementary material for: Acanthopanax senticosus total flavonoids alleviate lipopolysaccharide-induced intestinal inflammation and modulate the gut microbiota in mice
Source: Biosci Rep. 2022 Feb 7;42(2):BSR20212670. doi: 10.1042/BSR20212670 (PMC8821950; doi:10.1042/BSR20212670)
Supplement: Supplementary Data [file BSR-2021-2670_supp.pdf]

Dear editor,

Here are the full uncropped and unedited versions of the Western blots, and the outer edges of the membranes can be visible, but I am so sorry that I am unable to provide these Western blots that include the molecular mass markers. and that I have previously sent my explanations and specific steps to the editorial board. In addition, I will submit the specific actions I sent previously again as supplementary documentation.

Yours sincerely,

Xiaoya Wang

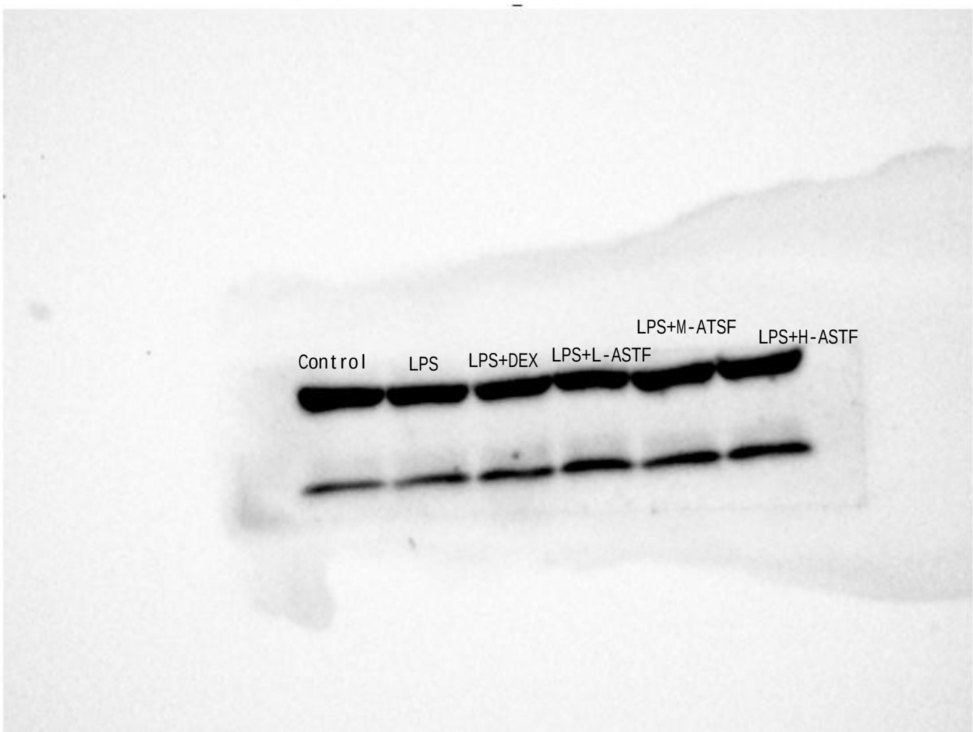

β-actin

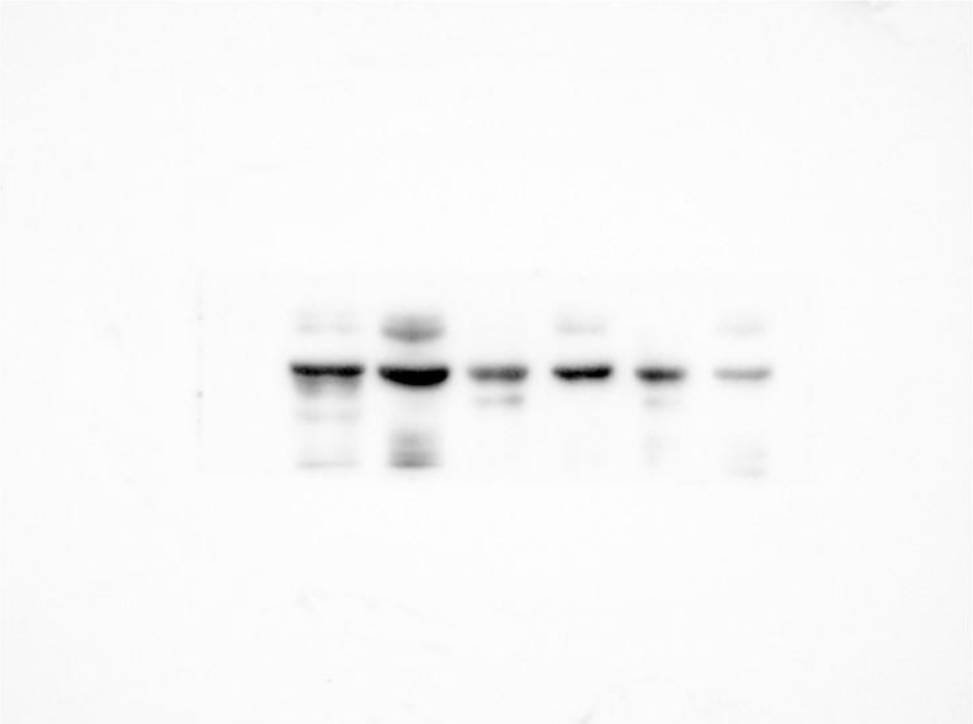

TLR4

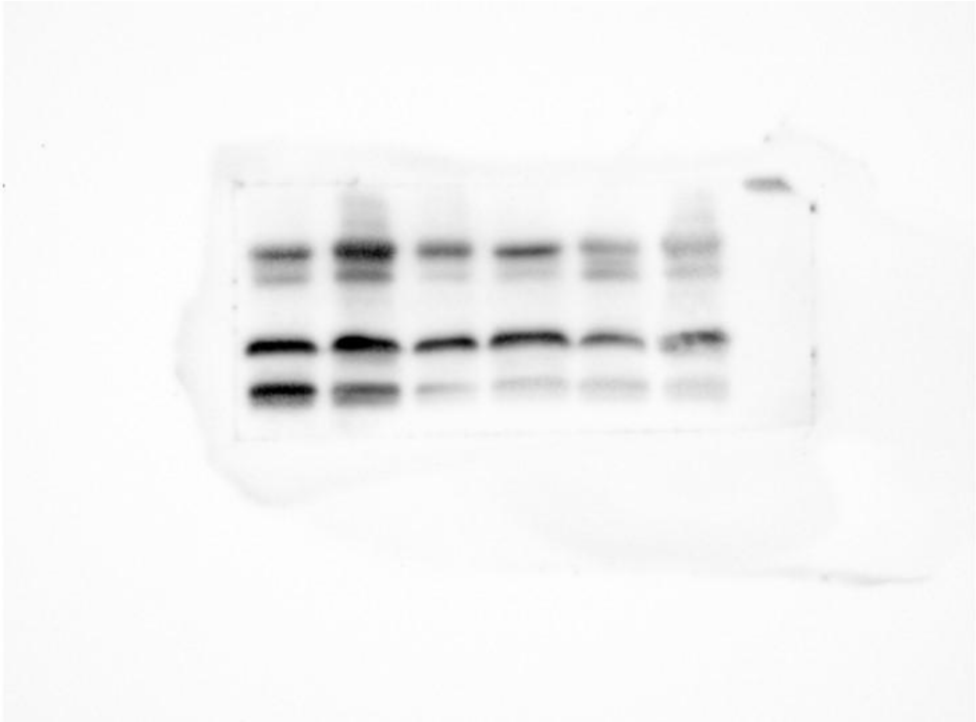

MyD88

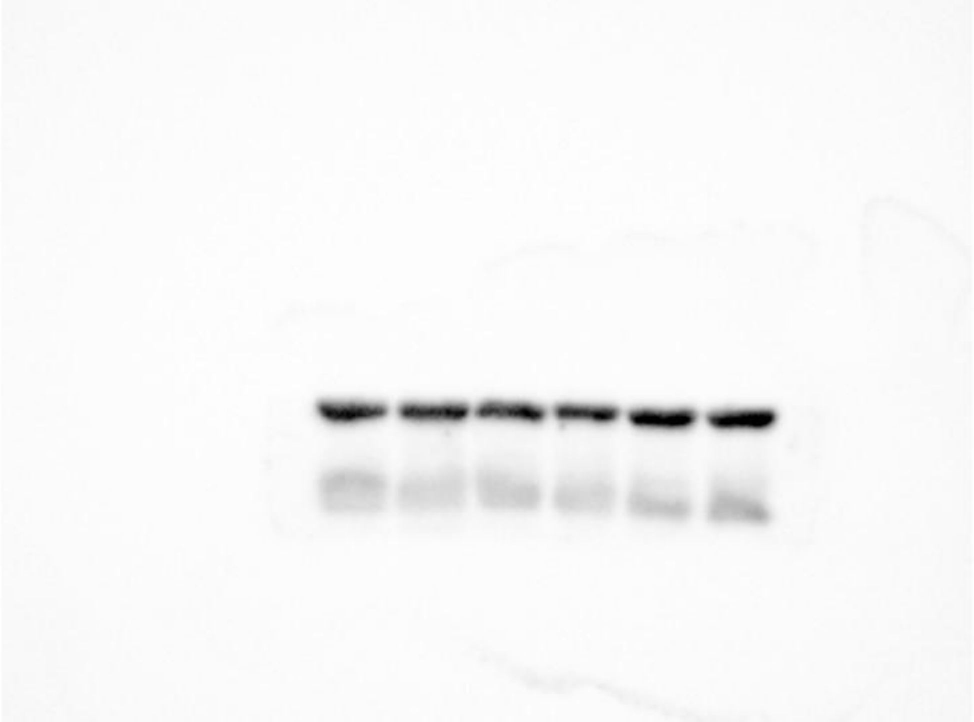

p65

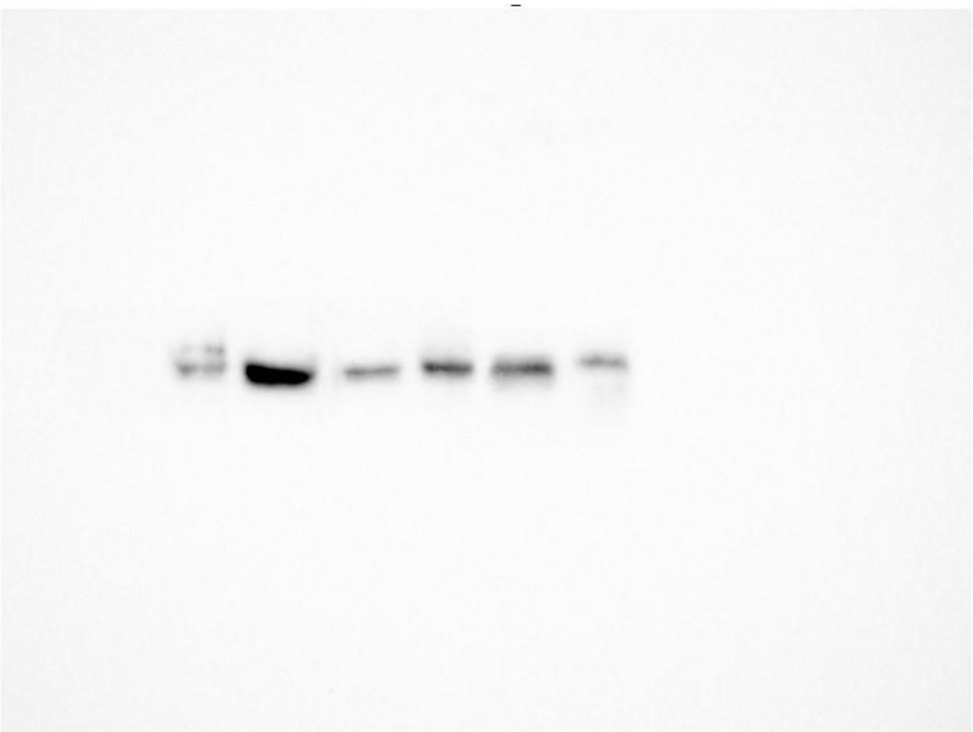

p-p65

The following is the imaging method and why there is no molecular mass markers:

1. Preparation of gel, (divided into concentrated gel and separating gel)
2. Insert the gel comb, and after the concentrated gel has solidified, remove the comb to form a swimming lane.
3. Add six sets of samples with the same amount of protein and a certain amount of marker.
4. The voltage of the concentrated glue is 60V, and the separation glue is 120V.
5. After the electrophoresis is over, judge the area where the target protein is located according to the molecular weight, cut the gel in the area where the target protein is located, and transfer it to the PVDF membrane for subsequent tests. Therefore, I cannot provide protein bands containing molecular mass markers. In addition, I made a figure to clarify my method of operation, I hope I make it clear. (If the entire gel is transferred to the PVDF membrane, the demand for PVDF membrane and antibody is too large to increase the experimental cost.)
6. Blocked for 3h, washed with TBST 3 times, 15min each time.
7. Incubated primary antibodies at 4°C overnight, wash 3 times with TBST, 15min each time.
8. Incubated secondary antibodies for 1.5h, wash 3 times with TBST, 15min each time.
7. Detected chemiluminescence by chemiluminescence imager with BeyoECL Star kit.
8. Take images with Bio-Rad gel imaging analysis system.

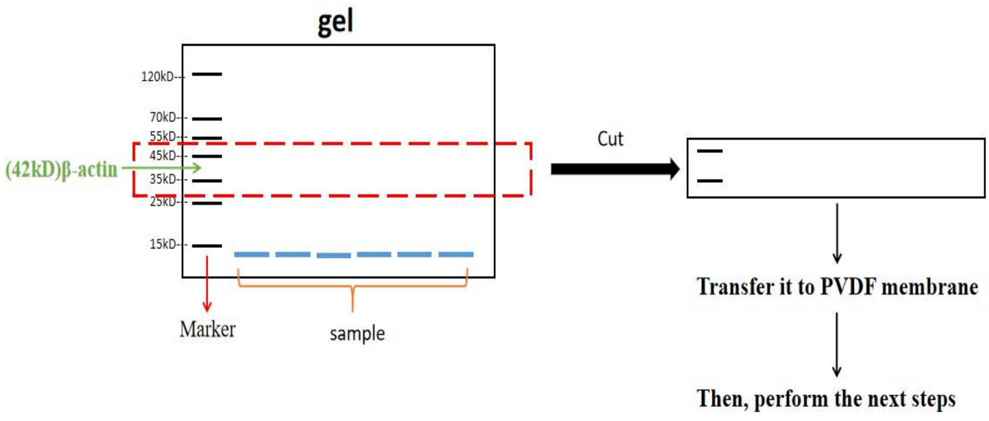

Take β-actin as an example
